# Neural model generating klinotaxis behavior accompanied by a random walk based on *C. elegans* connectome

**DOI:** 10.1038/s41598-022-06988-w

**Published:** 2022-02-23

**Authors:** Mohan Chen, Dazheng Feng, Hongtao Su, Tingting Su, Meng Wang

**Affiliations:** School of Electronic Engineering, Xidian University, Xi’an, 710071 China

**Keywords:** Computational neuroscience, Network models, Navigation, Computer modelling, Behavioural methods

## Abstract

Klinotaxis is a strategy of chemotaxis behavior in *Caenorhabditis elegans* (*C. elegans*), and random walking is evident during its locomotion. As yet, the understanding of the neural mechanisms underlying these behaviors has remained limited. In this study, we present a connectome-based simulation model of *C. elegans* to concurrently realize realistic klinotaxis and random walk behaviors and explore their neural mechanisms. First, input to the model is derived from an ASE sensory neuron model in which the all-or-none depolarization characteristic of ASEL neuron is incorporated for the first time. Then, the neural network is evolved by an evolutionary algorithm; klinotaxis emerged spontaneously. We identify a plausible mechanism of klinotaxis in this model. Next, we propose the liquid synapse according to the stochastic nature of biological synapses and introduce it into the model. Adopting this, the random walk is generated autonomously by the neural network, providing a new hypothesis as to the neural mechanism underlying the random walk. Finally, simulated ablation results are fairly consistent with the biological conclusion, suggesting the similarity between our model and the biological network. Our study is a useful step forward in behavioral simulation and understanding the neural mechanisms of behaviors in *C. elegans*.

## Introduction

*Caenorhabditis elegans* (*C. elegans*) is an ideal model organism for exploring the link between neural systems and behaviors. Its nervous system is compact, small in scale, easy to understand, and has been mapped almost entirely. An adult hermaphrodite contains only 302 neurons and over 7000 synaptic connections^1–3^. Furthermore, it exhibits a wealth of behaviors^4,5^ including orienting to a wide range of stimuli. Salt chemotaxis^6^, as a spatial orientation behavior, is fundamental for *C. elegans* survival because the bacteria on which it feeds release salt (NaCl) into the surrounding medium. Klinotaxis^6,7^ is a strategy of chemotaxis that gradually adjusts the direction of *C. elegans* towards a higher chemical concentration. This strategy relies on chemical differences between two sides of the body during locomotion. The head sensory neurons, ASEL and ASER, are mainly responsible for sensing NaCl^8,9^. However, because ASE neurons are too close together and *C. elegans* lies on its side, the spatial concentration information required for klinotaxis cannot be directly obtained from their responses. In fact, the ASEL/R neuron is an ON-/OFF-cell^9^, sensing the increase/decrease in NaCl at a single point. Therefore, responses of klinotaxis depend on both the sensory input and its current state during head sweeps dorsoventrally^10–12^. In addition, chemotaxis in *C. elegans* is irregular and obviously accompanied by a random walk. Random walk, as an unbiased exploration, exists during its locomotion, whether in an isotropic environment or in an environment with chemical gradients^7,13,14^. Researchers have found that chemotaxis strategies have little effect on the statistics of the random walk^14^, which preserves the ability to continuously explore environments in all directions and avoid local traps.

Despite the vast amount of available knowledge of behaviors and neural circuits and advancement in electrophysiology, the neural mechanisms underlying these behaviors are still not clearly delineated. The lack of information on neurophysiology data (such as strengths and polarities of synapses) is a major obstacle^15,16^. In this context, simulation models are useful in filling in the data gaps using optimization techniques. As a part of the experiment–theory cycle, they can provide validations or generate new hypotheses on the generation of behavior^16^.

Below, we review the related works on salt chemotaxis simulation models of *C. elegans*. Some early models attempted to predict the strategies^17^, computational rules^18^, and network connectivity patterns^19^ of chemotaxis. Subsequent models focused more on behavioral implementation. Xu et al*.*^20^ used dynamic neural networks to simulate salt attraction and toxin avoidance, and then incorporated body undulatory models to implement chemotaxis^21,22^. Demin et al*.*^23^ used a 3D simulator and an unrealistic neural circuit to control chemotaxis. Costalago-Meruelo et al*.*^24^ combined a spiking neural network with a physical engine to emulate chemotaxis. However, in the above models, either sensory models make the spatial concentration information directly available, or the chemotaxis processes do not actually follow klinotaxis. Izquierdo et al*.*^11^ evolved a minimal klinotaxis circuit, consisting of only two pairs of sensory and motor neurons, and revealed a state-dependent neural mechanism of klinotaxis; subsequently, the circuit was expanded to include two interneuron pairs^12,25^ and the same mechanism was found. Although the minimal model is too simplistic, it is a useful starting point for studying the neural model of klinotaxis in *C. elegans*. In addition, Zu et al*.*^26^ proposed a computational model which decomposed the sensory input into two concentration gradients respectively required for two strategies of chemotaxis and implemented it in a network model.

The realistic sensory response is a prerequisite for estimating plausible mechanism of klinotaxis. We note a recent electrophysiological report^27^ that stimulation with NaCl evoked all-or-none membrane depolarization in ASEL neuron. Previously, both ASEL and ASER were thought to be encoded by graded potentials, because studies have reported that ASER fails to generate Na^+^ action potential^28^. This characteristic of ASEL has not yet been constructed in these models. Further, most chemotaxis models ignore the random walk; for the few models^19,26^ that consider it, they directly added a random variable to motion outputs, which is biologically unrealistic. As yet no models have been reported in the literature that generate the random walk by the activities of neural networks.

Based on the above premises, we present a salt random-walk klinotaxis model of *C. elegans* to concurrently realize klinotaxis and random walk behaviors and explore the neural mechanisms of these behaviors. The model is constructed based on the connectome and functional neural circuits of *C. elegans* and evolved by a genetic algorithm (GA). Importantly, we incorporate the all-or-none depolarization characteristic of ASEL neuron and introduce the liquid synapse that we propose according to the stochastic nature of biological synapses into the model. Behavioral analysis verifies that the model can implement klinotaxis, which emerged spontaneously from network evolution. Through the analysis of sensorimotor transformation and neuronal dynamics, we identify the mechanism by which klinotaxis is generated in this model. In addition, we test the model in an isotropic environment, verifying that the neural network with liquid synapses is able to generate a random walk similar to that in real *C. elegans*. This provides a new hypothesis as to the neural mechanism underlying the random walk. Moreover, the model exhibited realistic klinotaxis accompanied by the random walk when tested in environments with NaCl gradients, just as real *C. elegans* does. Finally, we performed simulated ablation on neurons in the model to compare the similarity of the operation of our model to that of biological nervous system.

## Results

### Responses of ASE sensory neuron model to NaCl stimulation

To input realistic sensory responses into the klinotaxis model, we first constructed an ASE sensory neuron model (see “Methods”: ASE sensory neuron model), considering their electrophysiological characteristics recorded in previous studies^9,27–29^ and using a conductance-based approach^30^. We simplified each neuron to a single electrical compartment and required that the dynamics must meet the following characteristics. Regarding the ASEL neuron, (L1) it is an ON-cell, responding only to increases in NaCl concentration. (L2) The response is a stochastic all-or-none phenomenon; the greater the increase in concentration, the greater the probability of depolarization, but the depolarization amplitude remains the same. (L3) In response to a concentration step stimulus, the voltage rapidly rises to the peak and subsequently slowly decays to the resting state. Considering the ASER neuron, (R1) it is an OFF-cell, only responding to decreases in the NaCl concentration. (R2) The voltage response is in a graded manner. (R3) is the same as L3. (R4) The voltage response peak is a saturation function of the concentration decrease amplitude.

To verify whether the responses of the ASE neurons met the summarized characteristics, we applied various NaCl concentration step changes (up-/down-steps) to the neural network model (see “Methods”: Klinotaxis model: neural network model) to observe the responses of ASE neurons.

**Figure 1:**
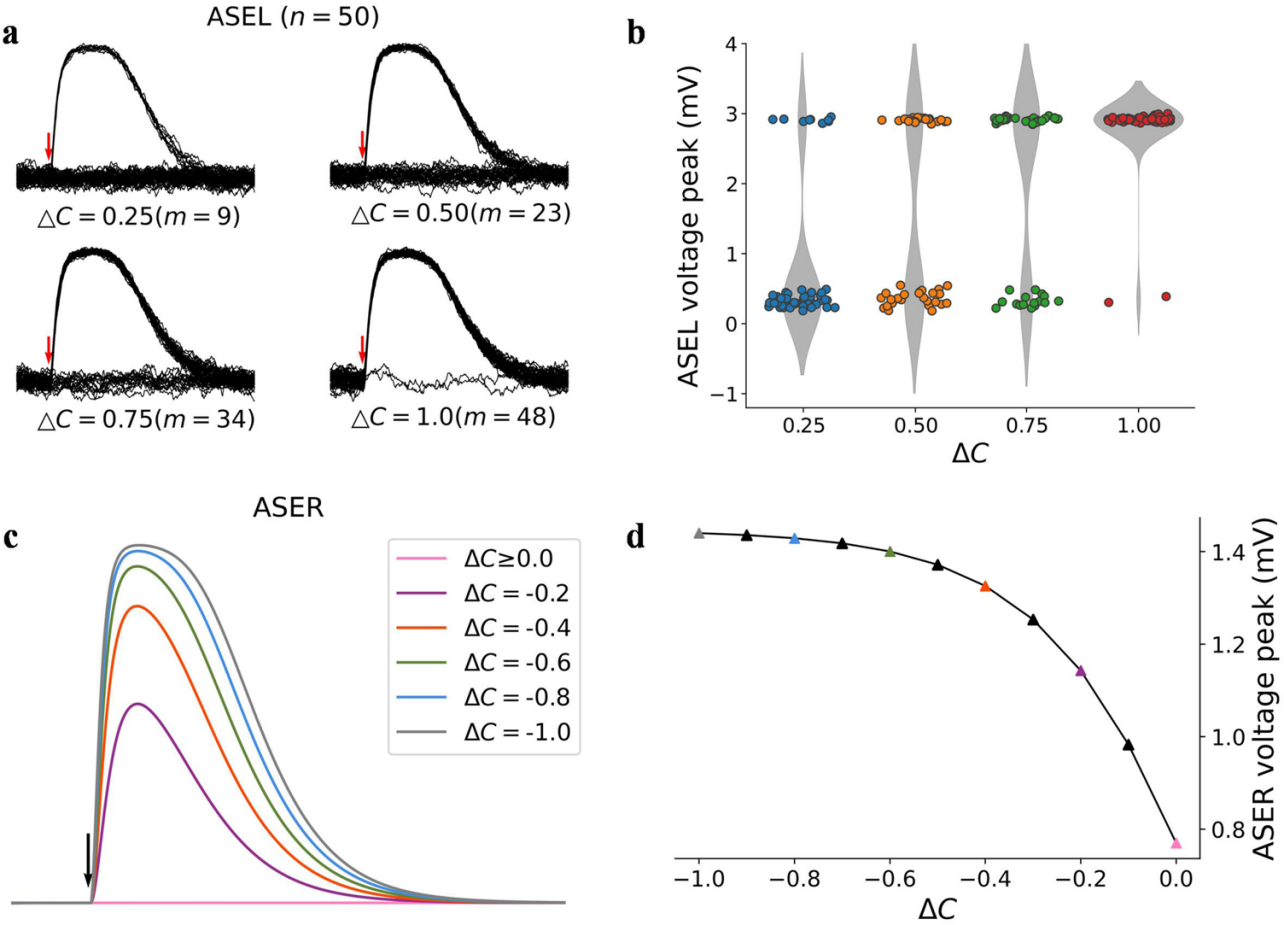
Voltage responses of ASE model evoked by up-/down-steps. (**a**) Voltage traces of ASEL neuron. *m* is the number of depolarizations evoked. (**b**) Voltage peaks of traces in (**a**), shown by colored dots. The gray areas are the violin plots, representing the kernel density estimations. (**c**) Voltage traces of ASER neuron. (**d**) The ASER voltage peak vs. down-step. The vertical arrows in (**a**), (**c**) indicate the onset times of stimuli.

The ON- and OFF-cells characteristics of ASEL and ASER (L1, R1) can be obtained from Eqs. (6) and (9). Figure 1a and b show the voltage dynamics of the ASEL neuron in response to up-steps. We performed multiple experiments (*n* = 50) for each up-step scenario, and added a small noise to the ASEL neuron to avoid a complete overlap of voltage traces. It may be observed that small up-steps evoked a small number of depolarizations; as the up-steps increase, the number increases. Considering the up-steps of different magnitudes, all the depolarized voltage peaks were similar. These indicate that ASEL had an all-or-none depolarization in response to NaCl stimulation as required in L2. During depolarization, the voltage trace underwent a process of rapid rise and subsequent slow decay, satisfying the characteristic in L3. The results reproduce the response pattern of ASEL neuron in biological experiments^27^.

Figure 1c and d show the voltage dynamics of the ASER neuron in response to down-steps. The voltage response peak increased with the increase in the magnitude of down-steps, that is, ASER had an obvious graded response, as required in R2. It may be observed from the shape of voltage traces that the dynamic satisfies R3. Moreover, the relationship between the ASER voltage peaks and magnitudes of down-steps, as shown in Fig. 1d, indicates a saturation relationship as required by R4. Therefore, the response dynamics of the ASER neuron satisfy characteristics summarized above.

### Klinotaxis behavior generated by the klinotaxis model

We constructed a connectome-based klinotaxis network model of *C. elegans* (see “Methods”: Klinotaxis model). The neurons in the neural network were chosen based on the functional neural circuits^31–34^ and the connection was based on the connectome^1–3^, involving chemical and electrical synaptic connections. The unknown parameters were evolved by a genetic algorithm (GA) for the purpose of achieving chemotaxis. Genetic algorithm generated a population of 300 models with fitness scores ranging from 0.26 to 0.79. Among them, there were 108 successful models with a fitness score higher than 0.7 and the *reliability* higher than 0.5 (see “Methods”: Terminology). Further analysis was limited to the successful sub-population. To verify whether these klinotaxis network models of *C. elegans* (hereinafter referred to as model *elegans*) followed klinotaxis, we tested them in environments with NaCl gradients and analyzed the behavior, referring to methods used for real *C. elegans*^7^ and the minimal klinotaxis model^11,12^. Definitions of variables in analysis refer to “Methods”: Terminology.

We first measured the chemotaxis performance in two environments (see “Methods”: Environmental models) to test the effectiveness and generalization of the successful models. In each time, these model *elegans* moved freely in different initial orientations at an initial position 4.5 cm away from the NaCl peak for 800 s and were executed 50 times in each environment. The results show that these model *elegans* achieved high chemotaxis indexes (*CIs*) and *reliabilities* regardless of the environment (Table 1).

**Table 1:** Chemotaxis performance of the successful model *elegans* sub-population in two environmental models (*n* = 108, ± s.e.m.).

Environmental model	Conical	Gaussian
*CI*	0.753 ± 0.0231	0.749 ± 0.0213
*Reliability*	0.996 ± 0.0383	0.990 ± 0.0962

**Figure 2:**
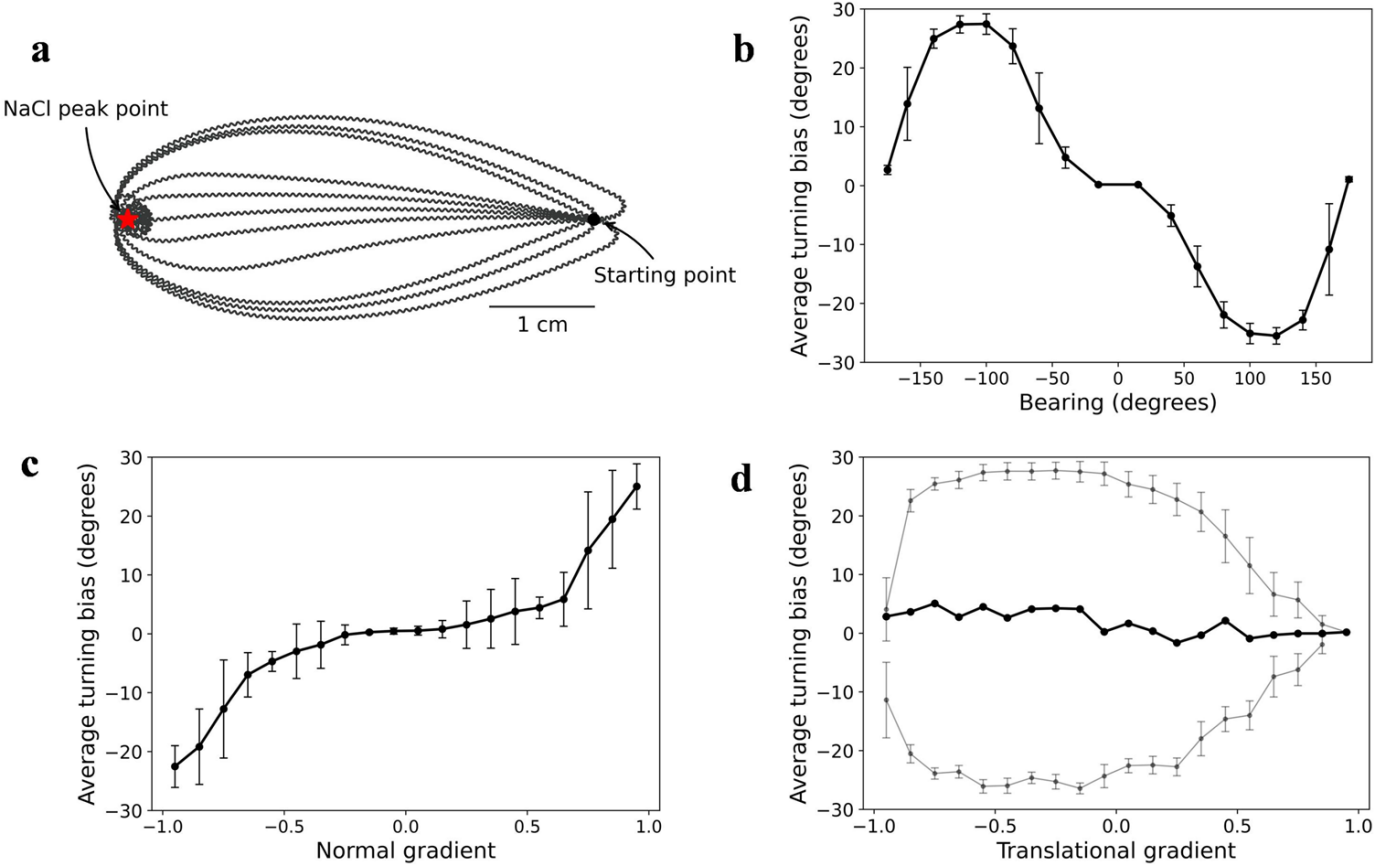
Behavioral analysis of klinotaxis in the model *elegans*. (**a**) Multiplication of the locomotion tracks with different initial orientations. (**b**) Average turning bias vs. bearing. (**c**) Average turning bias vs. normal gradient. (**d**) Average turning bias vs. translational gradient. The gray dots represent the average positive and negative turning biases. The error bars represent s.d.

Subsequently, we determined whether the behavior met klinotaxis. Considering a model *elegans*, its tracks are shown in Fig. 2a. The model *elegans* moved forward in a sinusoidal wave pattern and gradually adjusted its direction to the higher concentration side without sharp turns until it reached the NaCl peak. Based on the shape of the tracks alone, this behavior was consistent with klinotaxis in real *C. elegans*^7^. Then, we calculated and plotted the relationships between the turning bias and bearing, normal gradient, and translational gradient in tracks. The result is shown in Fig. 2b, c, and d, where gradients were linearly normalized to between -1 and 1. According to the judgment evidence of klinotaxis in real *C. elegans*^7^, (1) the relationship between the average turning bias and bearing should be approximately sinusoidal with the opposite algebraic signs; Fig. 2b shows that this was indeed the case. (2) The average turning bias should be proportional to normal gradient with the same algebraic signs; the relationship shown in Fig. 2c was consistent with this feature. (3) The average turning bias should be independent of translational gradient, which is reflected in Fig. 2d. However, if turning biases were divided into positive and negative, considering two translational gradient bins with the same value and opposite signs, the negative bin corresponds to greater turns. This suggests that the direction correction was greater when model *elegans* was far away from the NaCl peak, which has been found in the minimal klinotaxis model^12^. The above analyses indicate that the model *elegans* can realize klinotaxis.

Moreover, we performed the same quantitative behavioral analysis for 10 randomly selected model *elegans*, and the average results yielded the same conclusion (Fig. S1 in Supplementary Information 1).

### Sensorimotor transformation and mechanism underlying klinotaxis

**Figure 3:**
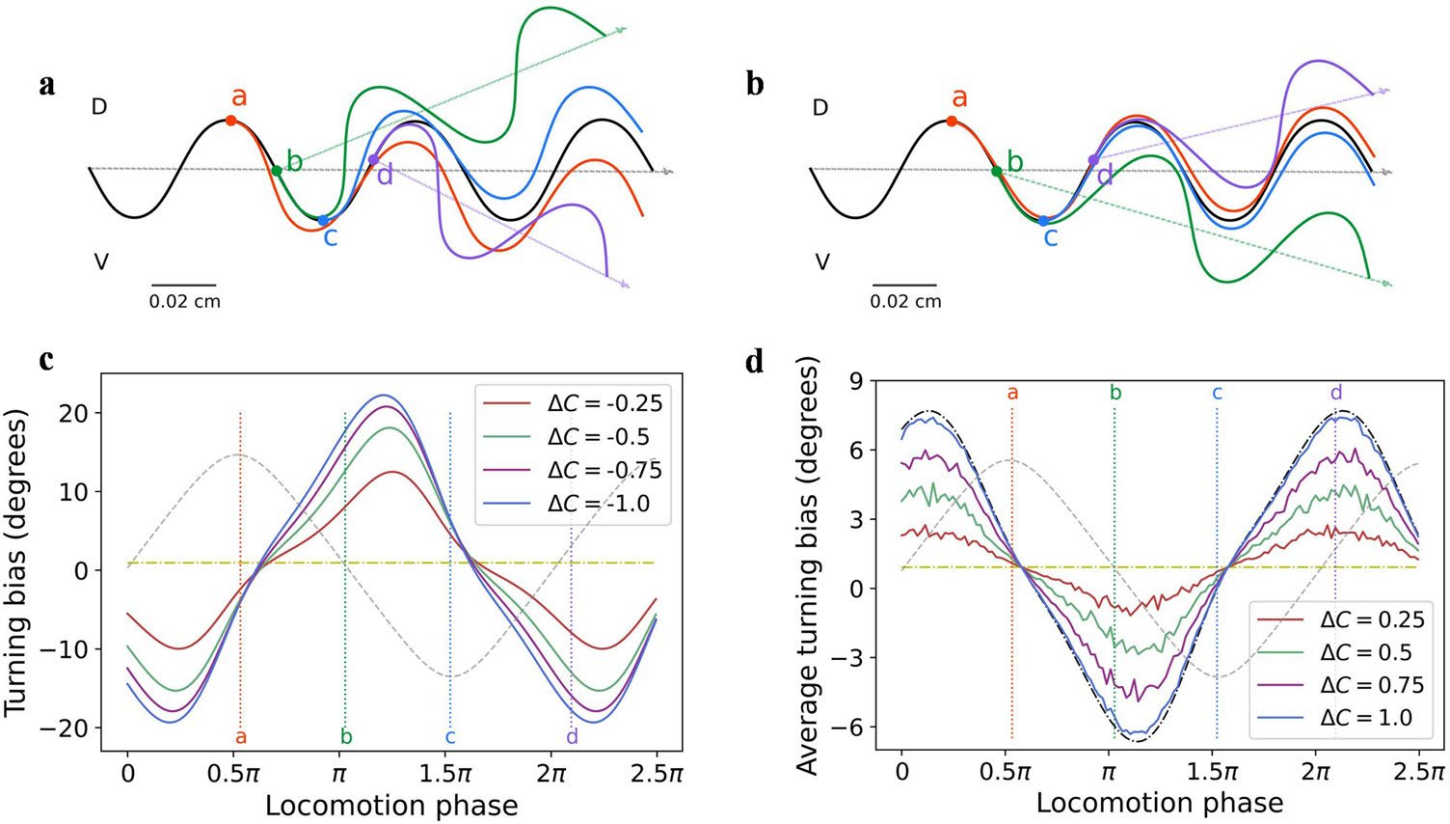
Steering responses of the model *elegans* elicited by up-/down-steps. (**a**), (**b**) Changes in locomotion track caused by down-steps (**a**) or up-steps (**b**). The black curves represent the tracks without stimuli, and the four colored curves respectively represent the tracks after applying an up-/down-step at the four phases of the corresponding colors. (**c**), (**d**) Steering response vs. locomotion phase at which a down-step (**c**) or an up-step (**d**) was applied. The four colored dotted lines represent the positions of four phases in (**a**) and (**b**). The gray dashed lines represent the shape of locomotion in all the phases. The yellow dash-dot lines represent the turning bias when ASEL was not activated or without stimulus. The black dash-dot line in (**d**) represents the turning bias when ASEL was activated.

To explore how the model generated klinotaxis, we analyzed sensorimotor transformation and neuronal dynamics of this model *elegans*. Figure 3 shows the sensorimotor transformation when up-/down-steps were applied at different locomotion phases. The steering response was quantified as the turning bias calculated over one cycle following a stimulus. Note that the ASEL’s responses to the up-steps were probabilistic and all-or-none; therefore, Fig. 3b only shows the case in which ASEL was activated, and Fig. 3d shows the average turning bias of multiple experiments (number is 100). The following conclusions can be drawn from the results in Fig. 3.

1. The down-/up-steps caused opposite steering responses. A down-step activated ASER, resulting in subsequent increases in the turning angle; as such, the translational direction was adjusted to the opposite side of the instantaneous direction of that phase (Fig. 3a). The reverse occurred when an up-step activated ASEL (Fig. 3b). These steering responses were effective because applying a down-/up-step implies that the instantaneous direction at that time is the direction of decreasing/increasing NaCl.
2. The steering response was related to the locomotion phase at which the stimulus was applied. As shown in Fig. 3a and d, at phase a/c, the translation direction was almost unchanged, because the component of the stimulus applied at this phase pointing to ventral/dorsal was approximately zero. At phase b/d, the adjustment amplitude was the highest, because the ventral/dorsal component of the stimulus applied at this phase was the largest. Moreover, the model *elegans* corrected its direction throughout the locomotion phase according to the discrepancies between its instantaneous direction and the direction of NaCl peak (Fig. 3c and d).
3. For a given phase, the amplitude of steering response was proportional to the amplitude of up-/down-steps (compare four solid lines in Fig. 3c or d); however, the formation mechanism was different. Down-steps caused graded responses of ASER, directly resulting in graded steering responses. In contrast, due to the all-or-none depolarization of ASEL, up-steps only resulted in two kinds of turning bias; the value when ASEL was not activated or activated is shown by the yellow or black dash-dot lines in Fig. 3d. The final average turning biases were distributed between these two; the larger the up-step, the greater the magnitude of average turning bias. This property allowed the model *elegans* to turn to a relatively better side when the polarities of the dorsal and ventral concentration changes were the same.
4. The steering response caused by the down-step was larger than that caused by the up-step with the same amplitude (compare *y*-axis values of Fig. 3c and d). This reflected the asymmetry of the gray dots shown in Fig. 2d at positive and negative translational gradients; that is, for the translational gradients with the same absolute value, the negative one induced a larger turning bias magnitude of model *elegans* than the positive. As such, the model *elegans* corrected the direction far away from the NaCl peak more strongly. This makes functional sense, allowing the model *elegans* to quickly correct deviations from the chemotaxis path while preventing oversteering.

The head dorsoventral sweeps during locomotion are critical to the klinotaxis strategy. In our model, the locomotion fluctuations were induced by sinusoidal oscillating signals ($I_{i}^{osc}$ in Eq. (12) in “Methods”) received by motor neurons; therefore, the sweeps were not spontaneously generated by the network. Nonetheless, the amplitude of fluctuations was determined through the evolution of the overall model, jointly influenced by $w^{osc}$, $w^{nmj}$, and parameters of motor neurons (refer to Eqs. (12) and (14) in “Methods”).

Subsequently, we analyzed how this sensorimotor transformation was formed by neuronal dynamics. The dynamics of sensory neurons have been given, and here we focus on motor neurons. In this model, the dynamic patterns of left and right motor neuron pairs are similar; therefore, only the left pair, SMBVL and SMBDL, are described as examples.

**Figure 4:**
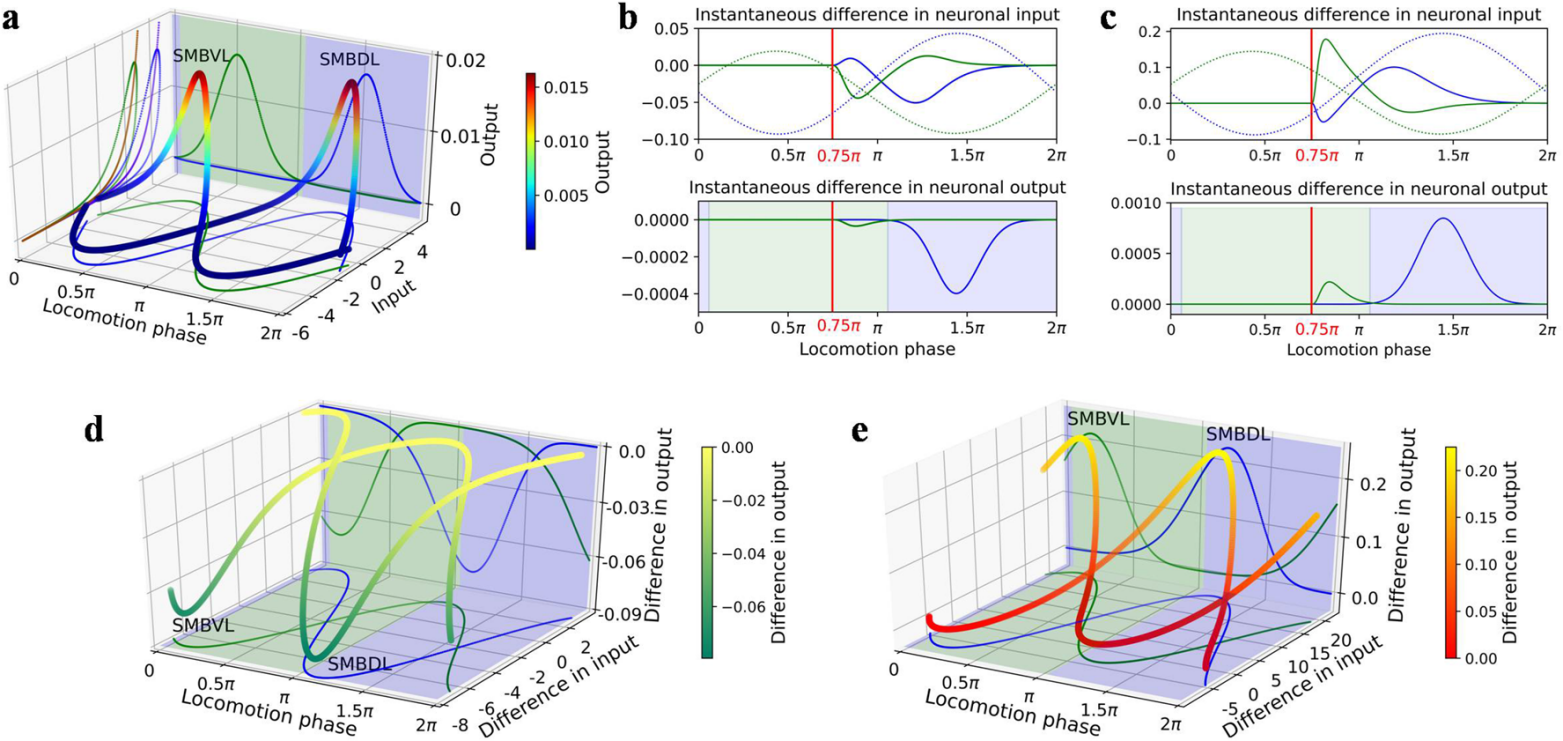
Dynamics of the motor neurons. Green and blue represent SMBVL and SMBDL respectively. The solid-colored curves in 3D sub-graphs show the projections of 3D curves onto each plane. The shaded areas represent the regions of the neuronal output sensitive to neuronal input in the absence of the sensory input. (**a**) Dynamics in the absence of sensory input. The brown and purple curves on the y–z plane show the SISO curves of the SMBVL and SMBDL, respectively. (**b**), (**c**) Instantaneous differences in the neuronal inputs and outputs induced by an up-step (**b**) or a down-step (**c**) at a phase of 0.75π. The dashed lines show the trends of the neuronal inputs with phase when there is no sensory input. (**d**), (**e**) Differences in the neuronal inputs and outputs induced by an up-step (**d**) or a down-step (**e**) at each locomotion phase.

The motor neuronal dynamics in the absence of sensory input, including the neuronal inputs, voltages, and outputs, are shown in Fig. 4a. When the oscillating input was omitted, the steady-state input–output (SISO) relationship was a nonlinear sigmoidal function, reflecting only one saturated region because of large negative biases of motor neurons (see brown and purple curves in Fig. 4a). When neuronal input was small, the output was in the saturated region where the output was insensitive to the input changes. In an actual locomotion, motor neurons received sinusoidal oscillating inputs ($I_{i}^{osc}$ in Eq. (12) in ”Methods”), making their instantaneous input–output states circulated counterclockwise around the SISO curves. The input of SMBVL and SMBDL varied sinusoidally with the locomotion phase throughout the locomotion cycle and were out-of-phase, causing SMBVL and SMBDL to enter alternately and do not occupy sensitive regions at the same time.

We then analyzed the resultant changes of dynamics in the presence of sensory input (for up-steps, only the case where ASEL was activated was considered). Figure 4b and c show the instantaneous differences of neuronal inputs and outputs in the subsequent period of time induced by an up-/down-step at the phase of 0.75π. An up-/down-step caused a continuous voltage response of ASEL/R, which was transmitted by interneurons, resulting in a continuous change in motor neuronal inputs and the change was bidirectional. However, owing to the influence of saturated region, the ASEL/R response only led to a decrease/increase in motor neuronal outputs. Considering the overall effect, when ASEL was activated, the inhibition of the SMBDL output was greater than that of the SMBVL output. Therefore, the model *elegans* veered toward the ventral side, and the ASER had an opposite effect. This explains the steering responses at the phase of 0.75π, as shown in Fig. 3.

We take the cumulative amount of instantaneous differences caused by a stimulus applied at a phase as the difference of this phase. Then the analysis was extended from 0.75π to all phases; the results are shown in Fig. 4d, e. For all phases, the ASEL/R response only led to a decrease/increase in motor neuronal outputs. SEMDL/VL outputs were alternately sensitive to sensory inputs; SMBDL was sensitive during ventral sweeps, whereas SMBVL was sensitive during dorsal sweeps. Consequently, during ventral sweeps, ASEL/R activation led to a decrease/increase in SMBDL output and a constant SMBVL output, resulting in a ventral/dorsal steering in model *elegans*, and the reverse was true for dorsal sweeps. This explains the direction of steering responses shown in Fig. 3. Moreover, comparing the magnitudes of output differences (i.e., the *z*-axis values shown in Fig. 4d and e), it can be observed that the amplitude of differences caused by the down-step were larger than that caused by the up-step. This explained the asymmetry of the turning bias amplitudes caused by down- and up-steps shown in Fig. 3c, d. Additionally, we showed the output differences of the SMBDL and SMBVL caused by the up- and down-steps in different sizes at each locomotion phase (see Fig. S2 of Supplementary Information 1), which more visually explained the steering response in Fig. 3c, d.

According to the above analysis, the mechanism of this model generating klinotaxis mainly depends on following principles. (1) The ASEL and ASER have antagonistic synaptic effects on motor neurons. This ensures that when the concentration changes in opposite polarity are sensed at the same locomotion phase, the opposite steering responses are caused. (2) Dorsal and ventral motor neurons are alternately sensitive to sensory input. This ensures that when the same concentration change is sensed during the dorsal and ventral sweeps, the opposite steering responses are caused, indicating state-dependence. (3) The sensory responses form graded average steering responses in different way, allowing the model *elegans* to turn to a more favorable side when the concentration changes during dorsal and ventral sweeps are of the same polarity. The mechanism which emerged in this model is approximately similar to that in the minimal klinotaxis model^11,12^. Nonetheless, due to the more realistic sensory coding pattern and network architecture, this model provides new findings as to the operating detail, such as ASEL forming the graded average steering responses by depolarization probabilities. In addition, in the minimal model^11,12^, the structure and parameters were set to be symmetrical across the dorsoventral midline to ensure that the communication of sensory neurons to bilateral motor neurons was symmetrical. In our model and the real nervous system, the connectome structure is not symmetrical. Our model spontaneously developed both the symmetry and mechanism during evolution, further demonstrating the plausibility of the mechanism in the biological network.

### Effect of the all-or-none depolarization characteristic of the ASEL neuron

The all-or-none depolarization characteristic of the ASEL neuron formed a decision-making capability that made the large and sustained concentration increases more likely to trigger responses. The resulting steering response to the up-step was all-or-none (fixed steering angle or no steering) in each locomotion phase; the graded average steering response to up-steps was formed by depolarization probabilities of the ASEL neuron. Consequently, the chemotaxis of the model *elegans* was achieved by combing the flexible steering in response to the down-step with the all-or-none steering in response to the up-step. It may make sense to allow for less energy consumption, and still having the flexibility to capture and turn in the favorable direction when *C. elegans* moves down gradients.

Subsequently, we analyzed how this characteristic affects the overall behavior during klinotaxis in the model. It can be observed from Fig. 2b that the average turning bias curve of model *elegans* was relatively flat when the bearing was near zero. Compared to the experimental data of real *C. elegans*^7^, the relationship curve between the average turning bias and bearing was not so sinusoidal. This is exactly due to the all-or-none characteristic of the ASEL neuron. When bearing had a small absolute value, there was a small deviation between the translational direction of model *elegans* and the direction to the NaCl peak. In this case, whether the head swept dorsally or ventrally, concentration gradients may be continuously positive and large; therefore, the ASEL neuron was highly likely to be activated during both sweeps. This reduced the turning amplitude of both side sweeps, resulting in no steering. Interestingly, it could be observed subsequently that when the behavior included the random walk, this deviation of relationship curve compared to that of real *C. elegans* was significantly improved (see Fig. 6f). Actually, considering the analyzed data for klinotaxis in real *C. elegans*^7^, researchers only removed the data corresponding to consecutive sharp turns, implying that the object analyzed was exactly the klinotaxis accompanied by the random walk. Therefore, the conclusion that the klinotaxis behavior with random-walk conforms to the real *C. elegans* data is more accurate and meaningful.

### Generalization and neuronal analysis

The result of the GA was not a unique model, but an ensemble of models with different parameter sets. We examined the network motif and neuronal parameter distributions of models in the successful sub-population. The mean and standard deviation (SD) of each parameter across these models were provided in Supplementary Information 2.

**Figure 5:**
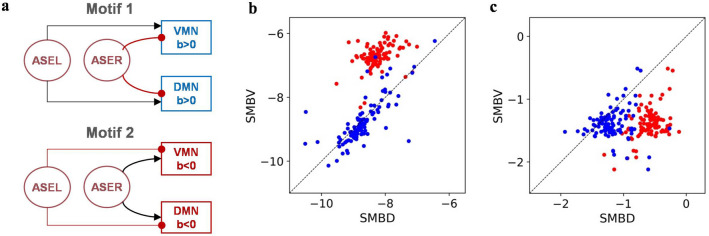
Klinotaxis network motifs and motor neuronal parameter analysis. (**a**) Two network motifs in minimal klinotaxis models^11^. Arrowheads, excitatory connections; filled circles, inhibitory connections. (**b**) Biases of motor neurons. (**c**) Steady-state voltages of motor neurons in the absence of the sensory and oscillating inputs. The blue dots represent the right pair of motor neurons and the red dots represent the left pair.

In the study of minimal klinotaxis models^11^, the evolved models were clustered into two network motifs, as shown in Fig. 5a. Analysis showed that all our evolved models were concentrated in Motif 2. That is, the biases of each motor neuron were negative with a large magnitude, as shown in Fig. 5b; ASEL and ASER neurons had inhibitory and excitatory synaptic effects, respectively, on motor neuronal outputs through complex interconnections of interneurons. This indicates that these models adopted the same mechanism analyzed in the previous section to implement klinotaxis. In the absence of sensory inputs, model *elegans* ran at as small a turning bias as possible until they sensed a change in the concentration and steered in an appropriate direction. This required that outputs of ventral and dorsal motor neurons varied with approximately the same amplitude in the absence of sensory inputs. Considering the right pair, ventral and dorsal motor neurons had fairly symmetric biases (Fig. 5b) and steady-state voltages (Fig. 5c); thus, when receiving oscillating inputs, their oscillating outputs were almost symmetric. Considering the left pair, the absolute values of negative biases of the dorsal motor neuron were always slightly greater than those of the ventral one. This can be compensated for by the different weights and reversal potentials of afferent neurons to them. The afferent neurons of the ventral motor neuron had a greater inhibitory effect on it; thus, its steady-state voltages in the absence of the sensory and oscillating inputs were always slightly lower than those of dorsal motor neuron, as shown in Fig. 5c. As such, the relative symmetry of the dorsal and ventral oscillating locomotion was ensured.

Our network models allowed us to explore the characteristics of interneurons in the realistic connection architecture. We mainly focused on neuronal biases, which affected the sensitivity of neuronal outputs to changes in inputs, and on the output changes of neurons in response to sensory inputs. Through analysis, we observed several interneurons which had relatively significant and stable characteristics. First, the magnitude of the biases of several interneurons, such as AIZR, AVBL, RIAL, RIAR and RIBR neurons, were consistently large in models; for sensory inputs in any direction (up- and down-steps), the output differences of these interneurons were little and negligible. Therefore, these neurons were insensitive to sensory inputs and were unimportant for klinotaxis in models. Considering these neurons with large magnitude biases (including motor neurons), their biases always tended to be negative, except for the RIBR neuron. This makes sense from the perspective of the energy constraint of nervous systems, because the negative bias saturates the synaptic conductance to zero.

Moreover, a critical interneuron, the AIZL neuron, was observed with biases steadily centered on the range of the sensitivity (mean 0.56, SD 0.49). The sensory inputs caused large output differences of the AIZL neuron. Furthermore, the ASEL and ASER sensory neurons had opposite synaptic effects on its output; the up-step increased its outputs, while the down-step decreased its outputs. This neuron had direct connections with the same polarity to the left pair of motor neurons. Thus, it can be concluded that the AIZL interneuron was important for the implementation of klinotaxis in these models. In addition, the AIBR neuron also had large and bidirectional output differences for sensory input in most models, with the up-step decreasing its outputs and down-step increasing its outputs; however, the variance of its biases was slightly larger across all models.

Additionally, there was a class of interneurons, AIYL and RIML neurons, whose biases were distributed in the range of sensitivity; nevertheless, in most models (AIYL: 87 out of 108; RIML: 101 out of 108), the synaptic effects from two sensory neurons had the same polarity. Sensory inputs induced only unidirectional changes in their outputs; both up- and down-steps leaded to an increase in outputs of the AIYL and RIML neurons.

### Liquid synapse and a hypothesis as to the neural mechanism of the random walk

Regardless of the chemical environment, the locomotion of *C. elegans* often involves the random walk^7,13,14^. The previous study^14^ has shown that chemotaxis strategies have little effect on the statistics of the random walk, which maintain its ability to continuously explore the environment in all directions, even when moving in the favorable direction. The study^35^ in *Drosophila larvae* has provided strong evidence that the random walk is generated intrinsically in the nervous system, without environmental interactions. However, it is not clear how this behavior is generated. In this study, we related statistical properties of the random walk to the variable nature of biological synapses.

According to the experimental data of real *C. elegans* in isotropic environments, the random walk has two behavioral features^7^. (W1) The curving rate varies randomly, and the overall distribution shows a normal distribution with a mean of zero and a SD of 32.3°/mm. (W2) The curving rate is strongly correlated with time, and the extent of the correlation changes over time. Distribution of curving rate is dependent on the previous 6 s curving rate (average value over 6 s is close to previous value), but independent of the previous 12 s curving rate (average value over 12 s is always zero).

Contrary to synapses in artificial neural network, the strengths of which are fixed after optimization, biological synapses are plastic and time-varying^36,37^. A major manifestation, long-term potentiation, has been recognized as the biological basis of learning and memory. In addition, the synapse is stochastic and variable to a certain extent due to many factors in the nervous system. For example, synapses are affected by transmitted signal noises such as various burst and indeterminate signals. Moreover, there are small noise sources in each synapse, which contribute to synaptic variability^37,38^, such as synaptic calcium channel noise, spontaneous activities of synaptic vesicles, and variability of receptor proteins in the postsynaptic membrane over time. These electrical and biochemical noises make synapses variable, thereby altering the amplitude of the postsynaptic current, and likely affecting information processing at macro level, such as behavior. According to the central limit theorem, when a phenomenon in nature is affected by many independent random factors, and if each factor makes a small impact, the total impact can be regarded as obeying a normal distribution.

Considering the above, we propose the concept of liquid synapse, and hypothesize that liquid synapses in the neural network can generate random walk behavior. The liquid synapse refers to the concept that the strength of the synapse varies slowly, randomly, and in small increments over time, and the overall distribution of each synapse follows the normal distribution with a small SD.

We introduced liquid synapses into the klinotaxis model to form the random-walk klinotaxis model of *C. elegans* (hereinafter referred to as random-walk model *elegans*). Each chemical synapse was assigned an independent normal distribution based on fixed parameters in the klinotaxis model. The mean of the distribution was determined through the evolution of klinotaxis function, analogous to long-term plasticity, i.e., the mean was the fixed synaptic strength in the klinotaxis model. This ensures that the klinotaxis function is not disrupted (i.e., liquid synapses cannot affect the plasticity outcomes related to learning). Considering the W2 feature of the random walk and the smoothness of the synapse, a random value in the corresponding distribution was assigned to the synaptic strength every 12 s. The synaptic strength varied linearly between consecutive guidepost values selected above. Thus, each synapse exhibited a continuous and slow variation over time. The SDs of the distributions, as a set of unknown parameters, were also optimized using the GA (cf. “Methods”: Klinotaxis model: model optimization). The fitness was evaluated by the similarity between the behavioral distribution of the model in an isotropic environment and the W1 behavioral feature.

Considering the network model with liquid synapses, the dynamic equation of neurons is consistent with Eq. (12) in “Methods”, except that chemical synaptic strengths are time varying. The equations of the liquid synaptic strength $w_{ij}^{c} \left( t \right)$ from neuron *j* to *i* are given as follows.

1 $$w_{ij,T}^{c} \left( k \right) \sim N\left( {\overline{w}_{ij}^{c} ,\sigma_{ij}^{2} } \right),$$

2 $$\left\{ {\begin{array}{*{20}l} {w_{ij}^{c} \left( t \right) = w_{ij,T}^{c} \left( k \right)} \hfill & {for\;\;t = k \cdot \Delta T + t_{0} } \hfill \\ {\frac{{dw_{ij}^{c} \left( t \right)}}{dt} = \frac{{w_{ij,T}^{c} \left( {k + 1} \right) - w_{ij,T}^{c} \left( k \right)}}{\Delta T}} \hfill & {for\;\;\left( {k \cdot \Delta T + t_{0} } \right) \le t < \left( {\left( {k + 1} \right) \cdot \Delta T + t_{0} } \right)} \hfill \\ \end{array} } \right.,$$

where *k* is the non-negative integer. $t_{0} \ge 0$ and $\Delta T = 12\;{\text{s}}$. To avoid confusion, the fixed chemical synaptic strength that evolved in the klinotaxis model is expressed as $\overline{w}_{ij}^{c}$ in this set of equations; $w_{ij,T}^{c}$ follows a normal distribution with a mean of $\overline{w}_{ij}^{c}$ and an SD of $\sigma_{ij}$.

### Realistic random walk generated by liquid synapses

**Figure 6:**
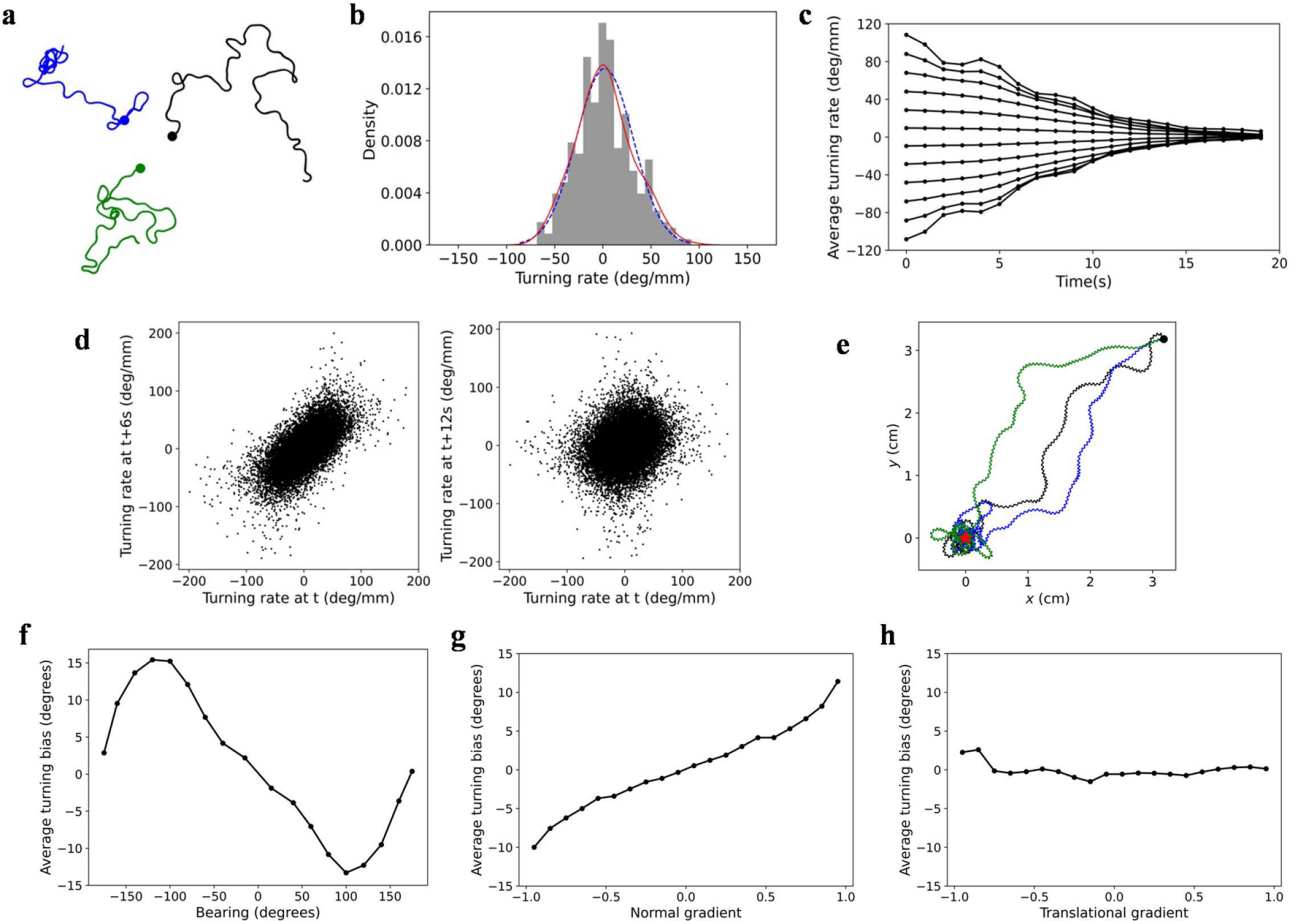
Behavioral analyses of the random-walk model *elegans* in an isotropic environment (**a**-**d**) and in an environment with NaCl gradients (**e**–**h**). (**a**) Three randomly selected tracks. (**b**) Distribution histogram of turning rates in a track (*μ* = 1.51 º/mm, *σ* = 30.52 º/mm, *P* = 0.38). The blue-dashed line is the probability density curve of the theoretical normal distribution. (**c**) Change in the average turning rates over time. (**d**) Relationship between turning rates at a time *t* and those after 6 s and 12 s. (**e**) Three randomly selected tracks in the presence of NaCl gradients. The black dot and red star represent the initial position and the NaCl peak, respectively. (**f**) Average turning bias vs. bearing. (**g**) Average turning bias vs. normal gradient. (**h**) Average turning bias vs. translational gradient.

To verify the above hypothesis, we tested the random-walk model *elegans* in an isotropic environment. In each experiment, the random-walk model *elegans* moved freely in a random initial orientation for 1,200 s, and the experiments were conducted 500 times. From the several tracks shown in Fig. 6a, the behavior was a diffused and undirected process which sometimes moved forward and other times circuitously, like the tracks of real *C. elegans*.

To verify whether the behavior met W1, a series of statistical analyses was performed on the turning rates in all tracks. We calculated the mean (*μ*) and SD (*σ*) of turning rates in each track. The average *μ* and *σ* were 1.593 ± 3.6790°/mm and 31.895 ± 2.3207°/mm (*n* = 500, ± s.d.), respectively, which were close to the values recorded in W1*.* In addition, the one-sample *Kolmogorov–Smirnov* (K-S) test was carried out on turning rates. This is a classical method for testing the goodness of the fit between sample and theoretical distributions. If *P* value is greater than the significance level (0.05), the null hypothesis that the sample conforms to theoretical distribution is not rejected. The average *P* value was 0.3679 ± 0.2696 (*n* = 500, ± s.d.), which was far beyond 0.05, indicating that turning rates obeyed the distribution required in W1. Figure 6b displays the distribution histogram of turning rates in one track, which visualized clearly that the distribution was approximate to the theoretical distribution.

To verify whether the behavior met W2, we analyzed the correlation over time of turning rates referring to the analysis method in biological experiments^7^. The turning rates were intercepted for consecutive 20 s starting with any initial value to form a strip. Then all strips were divided into bins based on their initial values. For each bin, we calculated the average once each second. As shown in Fig. 6c, each average turning rate was close to the value of previous moment; nonetheless, after approximately 12 s, the effect of the initial value almost disappeared, and the average turning rate in each bin tended to zero. Thereafter, we plotted the relationship between the turning rates at a time point and the turning rates after 6 s or 12 s (Fig. 6d). There was a significant positive correlation between the turning rates and the values after 6 s, but almost no correlation with the values after 12 s (the correlation coefficients were 0.674 and 0.182, respectively). These results are consistent with the W2 feature.

The above experimental analyses suggest that the random-walk model *elegans* is capable of generating the random walk similar to that in real *C. elegans*, confirming our hypothesis that liquid synapses in the neural network can generate the random walk.

### Realistic klinotaxis accompanied by the random walk generated by the model and complete chemotaxis behavior

Considering chemotaxis in real *C. elegans*, klinotaxis and random walk behaviors coexist. Therefore, we tested the random-walk model *elegans* by performing the same experiments as model *elegans* in environments with NaCl gradients. The chemotaxis performance is shown in Table 2. Influenced by the random walk, the average *CIs* were comparable with only a slight decrease compared to the model *elegans* and the *reliabilities* remained at a value of one. The tracks shown in Fig. 6e were not as neat as that of the model *elegans*; however, it eventually reached the NaCl peak and hovered around that point. These indicate that the random-walk model *elegans* was able to achieve chemotaxis accompanied by the random walk.

**Table 2:** Chemotaxis performance of the random-walk model *elegans* in two environmental models (*n* = 100, ± s.d.).

Environmental model	Conical	Gaussian
*CI*	0.712 ± 0.0355	0.701 ± 0.0371
*Reliability*	1.0	1.0

We performed the same verification method as adopted in the model *elegans* to verify whether the process conformed to klinotaxis. From the results shown in Fig. 6f, g, and h, we can observe the sinusoidal relationship between the average turning bias and bearing, the proportional relationship between the average turning bias and normal gradient, and the independence between the average turning bias and translational gradient. These relationships were consistent with the experimental results of real *C. elegans*, indicating that the model can realize klinotaxis accompanied by the random walk.

Chemotaxis in real *C. elegans* employs two strategies, klinotaxis^7^ and klinokinesis (biased random walk)^13^, which operate in parallel during chemotaxis. In contrast to the klinotaxis strategy which gradually adjust the direction of *C. elegans*, the biased random walk strategy reorients the locomotion through sharp turns. The biased random walk, based on the random walk, modulates the probability of sharp turns as a sigmoidal function of concentration temporal gradients^7,13^; for negative gradients, sharp turns occur more frequently, shortening runs down gradients. We included the biased random walk in the behavior generated by the random-walk model *elegans* to show the complete chemotaxis behavior of *C. elegans*. When the biased random walk strategy was implemented, the occurrence probabilities and turning angles of sharp turns were set based on the probability equation and turning angles given by Iino et al*.*^7^, which was obtained by fitting experimental data. We implemented the experiment 100 times and compared it with the random-walk klinotaxis behavior (see Fig. S3 in Supplementary Information 1). The results showed that when the biased random walk was included, the random-walk model *elegans* could better aggregate at the NaCl peak, which was consistent with the conclusion obtained by Iino et al*.*^7^.

### Results of the simulated ablation experiment

**Figure 7:**
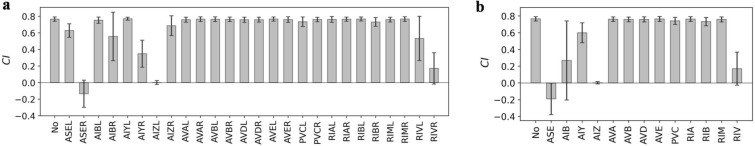
Klinotaxis efficiencies of multiple neuron-ablated random-walk model *elegans*. (**a**) Simulated ablation of an individual neuron. (**b**) Simulated ablation of a pair of neurons. The gray bar indicates the average *CI* of models, and the error bar is s.e.m. (*n* = 10).

In organisms, the effect of neurons on behavior can be explored by laser ablation. Similarly, we performed the simulated ablation experiment on evolved network models. We randomly selected 10 models from the successful sub-population and evolved their liquid synapses to obtain random-walk model *elegans*. Then, we conducted simulated ablations on neurons in each random-walk model *elegans* and measured the *CI*s to compare with the results of biological ablation experiments^7^. The average results are shown in Fig. 7.

Ablating the ASE pair led to a complete loss of klinotaxis; in this case, the model was unable to sense external stimuli. Ablating ASEL only slightly reduced the klinotaxis efficiency, whereas ablating ASER caused unstable klinotaxis defects. The biological ablation experiment^7^ has shown similar results, with ASER playing a major role and ASEL contributing to it; however, ASEL is not required.

In addition, biological experiment^7^ showed that although each interneuron has been implicated in chemotaxis in the studies of functional neural circuits^31–34^, for most interneurons, the ablation of a pair alone does not cause severe damage, except for AIZ. The simulated ablation of interneurons in our model yielded the similar results. Considering AIY or AIZ neuron pair, ablating AIYR or AIZL significantly affected klinotaxis, and the ablation of the other one (AIYL or AIZR) had no effect. However, ablating AIZ pair resulted in a severe impairment, whereas ablating AIY pair still allowed the model to perform klinotaxis with a slightly reduced efficiency. These are consistent with the biological conclusion^7^ that AIZ neurons are crucial to klinotaxis, whereas AIY neurons are not. Considering AIB neurons, ablating AIBR resulted in a decrease in performance; nevertheless, as can be seen by the variance, ablating AIBR had little effect in some models and serious effects in the other models. The same is true for ablating AIB pair. This is similar to the biological conclusion^7^ to some extent that ablating AIB neurons leads to a significantly decreased chemotaxis performance with a relatively large variance in the klinotaxis index of each tested *C. elegans.* The ablation of RIV neurons resulted in a significant defect in klinotaxis, which was the only inconsistency with the biological result. This can be explained by the oversimplification of the motor neurons, which in our model are only a group of SMB neurons. We selectively eliminated the connections of RIV and found that the RIV pair affected klinotaxis by directly innervating the SMBVL and did not affect other neurons. Thus, ablation of RIV resulted in a severe asymmetry of ventral and dorsal output. However, in real *C. elegans*, the head locomotion is controlled by multiple groups of motor neurons. Nonetheless, it should be noted that ablating RIV in real *C. elegans* eliminates the ventral bias of Ω-turns^31^. This can be indirectly reflected by our finding that RIV innervates ventral motor neuron SMBVL.

The fair consistency between the simulated ablation results of our model and the biological conclusion suggests that this model may operate through mechanisms similar to those in the nervous system. Both ablation experiments in the biological nervous system and the simulated ablation experiments in our model showed that the interneurons in the neural circuit were redundant for the klinotaxis behavior alone. Additionally, we constructed a random network with the same neurons and the same total number of connections, but the connection structure was randomly generated. Through evolving its parameters, this random network also realized the klinotaxis behavior (see Fig. S4 in Supplementary Information 1). Nevertheless, we do not suppose that these results deny the significance of the specific network structure of the nervous system. The behavior in *C. elegans* is complex and requires the compact nervous system to integrate a large amount of information with limited resources to generate appropriate and adaptive responses. In terms of the overall behavior, the specific network structure of *C. elegans* may be of great significance.

## Discussion

In this study, following knowledge of neuroanatomy, functional neural circuits, electrophysiology and behavior, we have presented a random-walk klinotaxis model of *C. elegans*. Simulation experiments have verified that the model can concurrently realize realistic klinotaxis and random walk behaviors. Through the model, we have identified a plausible neural mechanism underlying klinotaxis and provided a new hypothesized neural mechanism of the random walk.

Klinotaxis requires an interaction of the environment, nervous system, and behavior; thus we first constructed a sensory model. To our knowledge, this is the first implementation of an ASEL model with all-or-none depolarization, as well as the first salt chemotaxis model with two sensory coding patterns. In fact, in *C. elegans*, the orchestrated dynamics of two sensory neurons with different coding manners may be a common design to more efficiently integrate environmental signals to drive the appropriate behavioral outputs. For example, the chemotaxis for odorant diacetyl depends on both AWA coding positive gradients via stochastic pulsatile dynamics and AWC^ON^ coding negative gradients deterministically in a graded manner^39^. The deterministic response of one neuron is likely to compensate for the stochastic activity of the other to some extent, making behavior more robust. This sensory model allowed us to explore the mechanism of klinotaxis generation under the realistic sensory codes. In addition, our sensory model may be applied for other chemotaxis with similar sensory coding patterns.

Compared to the minimal klinotaxis model^11,12^, our model is closer to the biological network, both in terms of network architecture and sensory response characteristics. The neural mechanism underlying klinotaxis in our model is approximately similar to that in the minimal model. From the perspective of multiple roles of simulation models^16^, the significance is that the mechanism emerged spontaneously in a more realistic model, further demonstrating the plausibility of this mechanism and refining it. Furthermore, our model allows us to identify the characteristics of interneurons in the realistic network architecture.

Our model is the first to generate the random walk from activities of a neural network and generate klinotaxis accompanied by the random walk, just like the chemotaxis process in *C. elegans*. The liquid synapse we proposed is based on the nature of the real-world biological synapses, not our arbitrary thinking. From this we propose a new hypothesized mechanism whereby the liquid synapses affect behavior from the bottom up, generating the random walk. It is known that the sources of noise in nature are diverse; *C. elegans* is simultaneously affected by various internal noise from the nervous system and inevitably receives external sensory noise from the environment. When the sensory input noise is large enough, the behavior may be influenced and show randomness. Nevertheless, the random walk in *C. elegans* was often observed in petri dishes with less ambient noise. The study^35^ of *Drosophila larvae* with blocked synaptic activity in sensory neurons has shown that the random searching behavior can be spontaneously generated by the neural network independent of sensory system inputs. This strongly supports the view that the random walk is intrinsically generated by the nervous system. In a sense, this is reasonable. The random walk behavior preserves an exploratory searching ability of *C. elegans* to the environment; this searching ability should be intrinsic for organisms, rather than dominated by the environment. Additionally, in real nervous systems, random disturbances occur at multiple levels^37^, and the random walk may be caused not just at the synaptic level. Therefore, our hypothesis provides a plausible and not necessarily a unique solution to the way the nervous system generates the random walk behavior.

Further, we noticed two interesting phenomena. First, in the study of evidence for klinotaxis in *C. elegans*^7^, the data analyzed are actually the “run” part of locomotion, which include the random walk. Considering our model, compared to the klinotaxis behavior alone (Fig. 2b, c), the relationship curves of klinotaxis behavior accompanied by random walk (Fig. 5f, g) do resemble those of the previous study^7^. Second, the simulated ablation results are fairly consistent with the biological conclusion. These two phenomena were not designed specifically, but emerged spontaneously from the model. This suggests our model is effective and may operate through the mechanisms similar to the biological nervous system.

In our study, we evolved the klinotaxis and random walk behaviors sequentially using GA for simplicity reasons. Actually, in nervous systems, synaptic growth and learning occur in the presence of noise. That is, in biological synapses, the plasticity associated with learning is accompanied by noise-induced variability; these two are not separate. For the sake of biological plausibility, it can be considered to train all parameters simultaneously in one training in the future.

The simultaneous realization of both strategies of chemotaxis, the klinotaxis and biased random walk, in one model through plausible mechanisms is valuable and is a direction of our future research. We modified the neuron parameter to sufficiently increase the response amplitude of ASER neuron in our neural model; then, the model *elegans*’ steering response to negative gradients increased. When the magnitude of negative gradient was large enough, the trajectory formed a sharp turn. Combined with the random turning generated by liquid synapses, this resulted in a relatively high probability of sharp turns in negative gradients and a little probability of sharp turns in positive gradients. However, using biologically plausible neural mechanisms to well enough fit the function of the probability of sharp turns to temporal gradients of the concentration obtained in biological experiments, and to realize several types of behaviors of sharp turns, including short and long reversals that require backward movement, are needed to be further studied and extended on the basis of the current model. In addition, this study focused on the head navigation, and thus did not consider the body dynamics. The body and muscle models^40,41^ and mechanisms of undulatory locomotion^42,43^ of *C. elegans* should be combined in the future for further study of the overall locomotion during chemotaxis.

In general, our study is a useful step forward in the modeling-experiment cycle. It draws on available knowledge to simulate behaviors and in turn provides new insights into the neural mechanisms of behaviors in *C. elegans*. Furthermore, the realization of these behaviors in a simulation model provides theoretical support for worm-like navigation robotics systems.

## Methods

### ASE sensory neuron model

Considering the ASER neuron, the dynamics is expressed by an ordinary differential equation (ODE).

3 $$\tau_{R} \frac{{dV_{R} }}{dt} = - V_{R} - \sum\limits_{j} {w_{Rj}^{c} f\left( {V_{j} + b_{j} } \right)\left( {V_{R} - E_{Rj} } \right) - \sum\limits_{j} {w_{Rj}^{g} \left( {V_{R} - V_{j} } \right)} } - g_{R} \left( {V_{R} - E_{R}^{ext} } \right),$$

where subscript *R* and *j* correspond to the ASER neuron and neuron *j*, respectively. *τ* is the time constant, and *V* denotes the membrane voltage relative to the resting potential. The second and third terms are chemical and electrical synaptic currents from other neurons, respectively. $w_{Rj}^{c}$ and $w_{Rj}^{g}$ are chemical and electrical synaptic strengths from neuron *j* to ASER, respectively. $f\left( \cdot \right)$ is the sigmoid function, which expresses the nonlinear effect of presynaptic voltage on the postsynaptic voltage. *E* denotes the reversal potential and *b* denotes the bias. The last term represents chemosensory input current, where $E_{R}^{ext}$ is the equilibrium potential and $g_{R}$ represents the conductance of ion channels that transmit the stimulus signals. When there is no external stimulus, channels are closed by default, and the state is regulated by a pair of simple state-transition equations as follows.

4 $$\frac{{du_{R} }}{dt} = - \alpha_{R} u_{R} + \beta_{R} h_{R},$$

5 $$\frac{{dh_{R} }}{dt} = \alpha_{R} u_{R} - \beta_{R} h_{R},$$

where $\beta_{R}$ is a free parameter, and $h_{R}$ is a variable that directly controls the conductance of channels. $\alpha_{R}$ is a variable affected by external stimuli.

6 $$\left\{ {\begin{array}{*{20}l} {\alpha_{R} = - \Delta C} \hfill & {{\text{if}}\;\;\Delta C < 0} \hfill \\ {\tau_{\alpha R} \frac{{d\alpha_{R} }}{dt} = - \alpha_{R} } \hfill & {{\text{otherwise}}} \hfill \\ \end{array} } \right.,$$

where $\tau_{\alpha R}$ is the decay time constant of $\alpha_{R}$. The real ASE neurons are adaptive and adjust their sensitivity to respond to shallow or large concentration changes^44^. Here, we assume that ASE neurons have adapted to the current environment, and the chemosensory signal $\Delta C$ received by ASE is the instantaneous change in concentration scaled to between −1 to 1. Finally, conductance is derived by

7 $$g_{R} = \tanh \left( {\gamma_{R} h_{R} } \right),$$

where the constant parameter $\gamma_{R}$ combined with the hyperbolic tangent function (tanh) ensures the saturation response property of the ASER neuron.

Contrary to the ASER model, the ASEL model depolarizes in an all-or-none manner. The dynamic equation is as follows.

8 $$\tau_{L} \frac{{dV_{L} }}{dt} = - V_{L} - g_{L} \left( {V_{L} - E_{L}^{Ca} } \right),$$

where the subscript *L* corresponds to ASEL. The voltage of the ASEL model is determined by calcium ion channels, because EGL-19, a type of voltage-gated calcium channels, was found to be essential for the depolarization of ASEL^27^. $E_{L}^{Ca}$ is the equilibrium potential of calcium ions, and $g_{L}$ represents the conductance which controls the state of channels. The channels are activated when ASEL senses an increase in the NaCl concentration and the integrated afferent signal exceeds a threshold. The state-transition equations are the same as those of ASER, except that $\alpha_{L}$ is set as

9 $$\left\{ {\begin{array}{*{20}l} {\alpha_{L} = 1} \hfill & {{\text{if}}\;\Delta C > 0\;{\text{and}}\;in_{L} > in_{thres} } \hfill \\ {\tau_{\alpha L} \frac{{d\alpha_{L} }}{dt} = - \alpha_{L} } \hfill & {{\text{otherwise}}} \hfill \\ \end{array} } \right.,$$

where $\tau_{\alpha L}$ is the decay time constant of $\alpha_{L}$, and $in_{thres}$ is the threshold. $in_{L} = \Delta C + net_{L} + \xi_{L}$ is the integrated afferent signal of ASEL, where $net_{L}$ is the afferent signal from other neurons, including the chemical and electrical synaptic signals limited by a tanh function.

10 $$net_{L} = \tanh \left( { - \sum\limits_{j} {w_{Lj}^{c} f\left( {V_{j} + b_{j} } \right)\left( {V_{L} - E_{Lj} } \right)} - \sum\limits_{j} {w_{Lj}^{g} \left( {V_{L} - V_{j} } \right)} } \right).$$

The mechanism of the stochastic depolarization of the ASEL neuron is still unclear. To make the response satisfy this characteristic, we assume a random signal $\xi_{L}$ that varies uniformly and randomly within a certain range. Finally, the conductance is denoted as

11 $$g_{L} = \tanh \left( {\gamma_{L} h_{L} } \right),$$

where $\gamma_{L}$ is a constant combined with the tanh function to ensure the same depolarization amplitude.

In real nervous system, ASE neurons have synaptic connections from other neurons^1^; therefore, this ASE model considers afferent signals from other neurons, which has been ignored in most previous models.

### Klinotaxis model: neural network model

**Figure 8:**
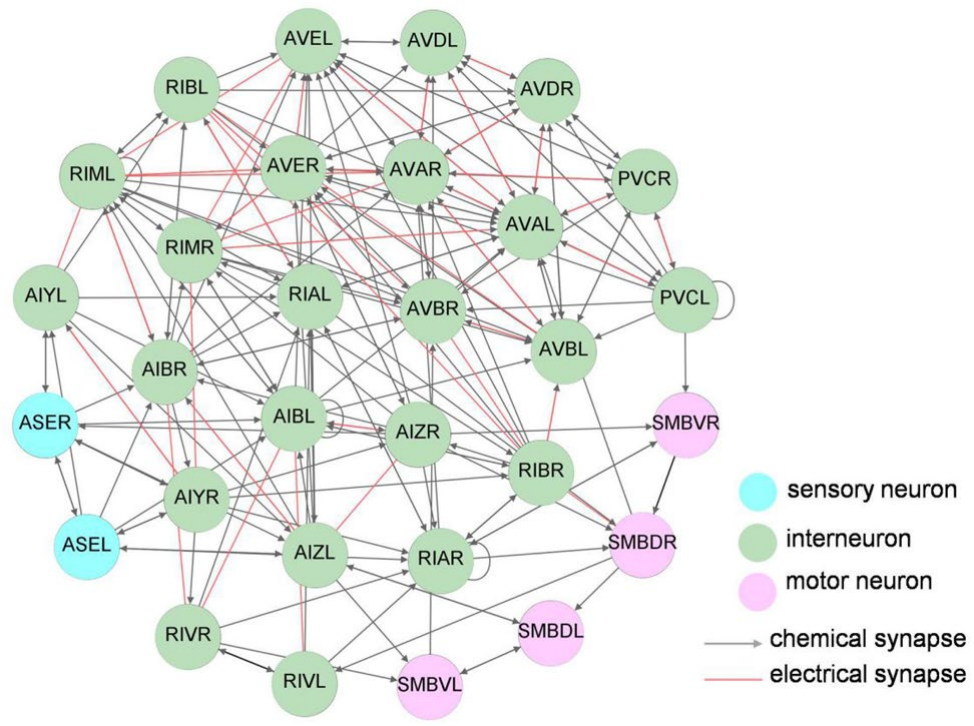
Architecture of the neural network model regarding chemotaxis in *C. elegans.*

The klinotaxis neural network architecture, as shown in Fig. 8, is constructed based on the functional neural circuit^7,31–34^ and neuroanatomical data^1–3^ of *C. elegans*. This network consists of 12 pairs of interneurons. Among them, RIM neurons (originally known as motor neurons) have been redistributed as interneurons^3^ and RIV neurons are polymodals that can be regarded as either interneurons or motor neurons^3^. Excluding RIA and AVE neurons, the remaining interneurons, which were identified to contribute to modulating the locomotion underlying chemotaxis, were summarized by Kocabas et al.^33^ according to previous assays about functional neural circuits^7,31,32^. In recent years, the sensory encoding of RIA interneurons^34^ was found to be gated according to head orientation, which may involve attention to asymmetric stimuli during head sweeps. The AVE neurons are the command interneurons which are found to generally have more synaptic connections and directly synapse to motor neurons to promote forward or backward locomotion^1,32^; in particular, AVE neurons have rich reciprocal connections with other command interneurons included in the model (AVA, AVB, AVD, and PVC). Additionally, SMB motor neurons have been identified to set the amplitude of sinusoidal undulations^31^. This may influence the gradual turning underlying klinotaxis. Together with the chemosensory neurons ASE, they form the klinotaxis neural network. The connections between neurons follow the connectome of *C. elegans*^1–3^, involving chemical and electrical synaptic connections.

Although some neurons in *C. elegans* have been observed to cause action potentials in recent years^45,46^, most neurons are still thought to exhibit graded potentials^28^. Therefore, the neurons (except ASE) in the model are represented by ODEs with graded potentials. The voltage dynamic equation of neuron *i* is given as follows.

12 $$\tau_{i} \frac{{dV_{i} }}{dt} = - V_{i} - \sum\limits_{j} {w_{ij}^{c} f\left( {V_{j} + b_{j} } \right)\left( {V_{i} - E_{ij} } \right) - \sum\limits_{j} {w_{ij}^{g} \left( {V_{i} - V_{j} } \right)} + \delta_{i} I_{i}^{osc} } .$$

Excluding the last term, the rest is the same as the dynamic equation of the ASER neuron and the symbols have the same meaning; hence, they are not repeated here. $w_{ij}^{c} \ge 0$, $w_{ij}^{g} \ge 0$ and $w_{ij}^{g} = w_{ji}^{g}$. Since *C. elegans* moves in a sinusoidal wave pattern, a sinusoidal oscillator component is used to generate the oscillating signal, and the last term represents the input from it. $\delta_{i} = 1$ for the motor neurons and $\delta_{i} = 0$ for the others. $I_{i}^{osc} = w^{osc} \sin \left( {{{2\pi t} \mathord{\left/ {\vphantom {{2\pi t} {T^{osc} }}} \right. \kern-\nulldelimiterspace} {T^{osc} }}} \right)$ with $T^{osc} = 4.2{\kern 1pt} \;{\text{s}}$ for one cycle of the sinusoidal locomotion^18^, and $w^{osc} > 0$ is the connection strength from the oscillator. The dorsal and ventral motor neurons receive oscillating input in the opposite phases.

### Klinotaxis model: kinematic model

The kinematic model refers to that in the minimal klinotaxis model^11,12^. The model is treated as a point with position coordinates (*x*, *y*) on a two-dimensional (2D) plane. The position is updated as follows.

13 $$\left( {\frac{dx}{{dt}},\frac{dy}{{dt}}} \right) = \left( {v\cos \left( \theta \right),v\sin \left( \theta \right)} \right),$$

where $v = 0.022$ cm/s is the constant speed based on measured data^18^ and *θ* is the instantaneous angular direction.

According to graded synaptic transmission at the neuromuscular junction (NMJ)^47^ and two assumptions^11^, the turning angle *φ* is controlled by motor neuron outputs $f\left( {V_{i} + b_{i} } \right)$.

14 $$\varphi = \frac{d\theta }{{dt}} = w^{nmj} \left( {\sum\limits_{{i_{{\text{D}}} }} {f\left( {V_{{i_{{\text{D}}} }} + b_{{i_{{\text{D}}} }} } \right)} - \sum\limits_{{i_{{\text{V}}} }} {f\left( {V_{{i_{{\text{V}}} }} + b_{{i_{{\text{V}}} }} } \right)} } \right),$$

where the subscripts $i_{{\text{D}}} \in \left\{ {\text{SMBDL,SMBDR}} \right\}$ and $i_{{\text{V}}} \in \left\{ {\text{SMBVL,SMBVR}} \right\}$ represent dorsal and ventral motor neurons, respectively. $w^{nmj} > 0$ is the connection strength of the NMJ.

### Klinotaxis model: model optimization

The unknown parameters were optimized by a genetic algorithm (GA)^48^, which simulates the process of natural evolution to search the optimal solution. Here, a total of 463 parameters to be optimized (including ${\mathbf{w}}^{c}$, ${\mathbf{w}}^{g}$, **E**, **b**, $E_{L}^{Ca}$, $E_{R}^{ext}$, $w^{osc}$, $w^{nmj}$) were randomly encoded as real-values between 0 and 1, forming an individual. 300 individuals constituted a population of the initial generation. Considering each generation, crossover, mutation, and selection operations were performed. The crossover rate was 0.8. When performing the crossover, two individuals were randomly selected as parents. Five new individuals were spawned evenly along the direction of their connecting line. We evaluated each one using the fitness function and only the highest scoring individual was retained as a child. The mutation rate was 0.3. When performing the mutation, a Gaussian noise with a mean of zero and a standard deviation of 0.05 was added to the child. If the fitness score of the child is better than that of one parent, the child is substituted for the lower scoring parent. Iteration stopped if 1000 generations were reached or if the fitness scores of the best individual did not improve significantly for 50 consecutive generations.

The fitness function is defined as the average chemotaxis index (*CI*) of multiple experiments. The *CI* is determined by the time average of the distance between the klinotaxis model and the NaCl peak point during the whole locomotion.

15 $$CI = 1 - \frac{1}{T}\int_{0}^{T} {\frac{r\left( t \right)}{{r\left( 0 \right)}}dt} ,$$

where *r*(*t*) is the Euclidean distance to the NaCl peak point at time *t*, and *r*(0) is the initial distance. *T* is the total time in one experiment.

### Environmental models

To optimize the model and test its generalization, two NaCl environmental models were used. The first is a conical gradient model used in the minimal klinotaxis model^11,12^, in which the concentration gradient is independent of the spatial location. The concentration C(*t*) at time *t* is linearly proportional to the Euclidean distance from the NaCl placement point.

16 $$C\left( t \right) = \kappa \sqrt {x\left( t \right)^{2} + y\left( t \right)^{2} } ,$$

where $\kappa$ is the conical gradient coefficient. We apply this environment in the context of optimization.

The other is a Gaussian-shaped gradient model used in classical *C. elegans* chemotaxis assays^7^. $C\left( t \right)$ is calculated using the Fick’s equation with 2D diffusion with no border.

17 $$C\left( t \right) = \frac{{N_{0} }}{{4\pi d_{c} D_{c} \left( {t + t_{0} } \right)}}\exp \left( { - \frac{{r^{2} }}{{400D_{c} \left( {t + t_{0} } \right)}}} \right),$$

where *N*_0_ is the NaCl solution concentration. *D*_*c*_ is the diffusion coefficient, and *d*_*c*_ is the agar plate thickness. *t*_0_ is the diffusion time before experiments, and *r* is the distance from the NaCl placement point. This environment is used to test the generalization of the model.

### Terminology

*Reliability* refers to the proportion of *C. elegans* that can reach NaCl peak point (in our simulations, a circular area within 0.1 cm of the peak point) in all experiments. The *turning bias* is the sum of the turning angle *φ* in one sinusoidal cycle. The *instantaneous direction* is the locomotion direction at the current moment. The *translational direction* is defined as the connecting direction of two positions that differ by one sinusoidal cycle, which is equivalent to the forward direction of *C. elegans* in one cycle. The *normal direction* refers to the direction in which the translational direction is rotated 90° counterclockwise. The *translational gradient* is the concentration gradient in the translational direction. The *normal gradient* is the concentration gradient in the normal direction. *Bearing* is the angle between the translational direction relative to the direction of the NaCl peak point. The *turning rate* is defined as the turning bias per unit translational distance.

## Supplementary Information

Supplementary Information 1.

Supplementary Information 2.
