# Supplementary material for: Neural model generating klinotaxis behavior accompanied by a random walk based on C. elegans connectome
Source: Sci Rep. 2022 Feb 23;12:3043. doi: 10.1038/s41598-022-06988-w (PMC8866504; doi:10.1038/s41598-022-06988-w)
Supplement: Supplementary file 1 — Supplementary Information 1. [file 41598_2022_6988_MOESM1_ESM.pdf]

Supplementary Information 1

Neural model generating klinotaxis behavior accompanied by a random walk based on *C. elegans* connectome

Mohan Chen[1], Dazheng Feng[1,*], Hongtao Su[1], Tingting Su[1] & Meng Wang[1]

[1]School of Electronic Engineering, Xidian University, Xi'an, 710071, China
[*]dzfeng@xidian.edu.cn

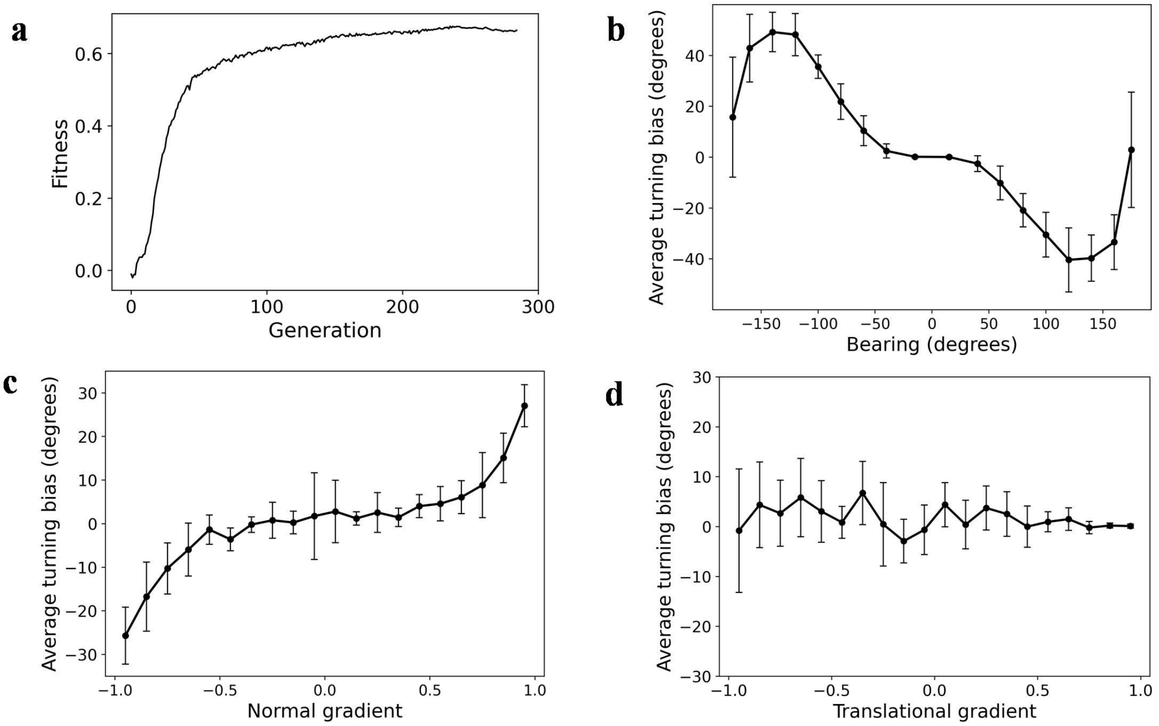

Figure S1. Convergence curve of the population evolved by the genetic algorithm (GA) and behavioral analysis of klinotaxis in multiple model *elegans*. (**a**) Fitness convergence curve. (**b**) Average turning bias vs. bearing. (**c**) Average turning bias vs. normal gradient. (**d**) Average turning bias vs. translational gradient. The error bars represent s.e.m. (n=10)

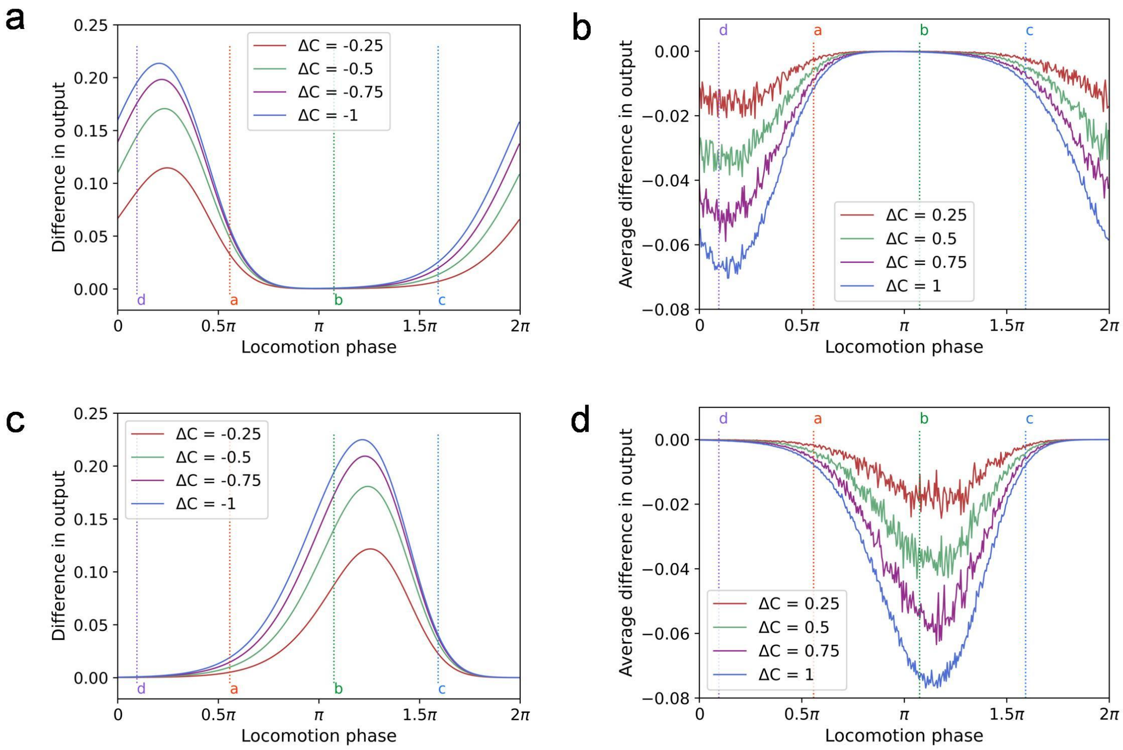

Figure S2. Output differences of SMBVL and SMBDL motor neurons induced by different size concentration step changes at each locomotion phase. (**a**) Differences in the SMBVL output caused by different down-steps. (**b**) Average differences in the SMBVL output caused by different up-steps. (**c**) Differences in the SMBDL output caused by different down-steps. (**d**) Average differences in the SMBDL output caused by different up-steps. The four colored dotted lines represent the positions of four representative phases in Fig. 3.

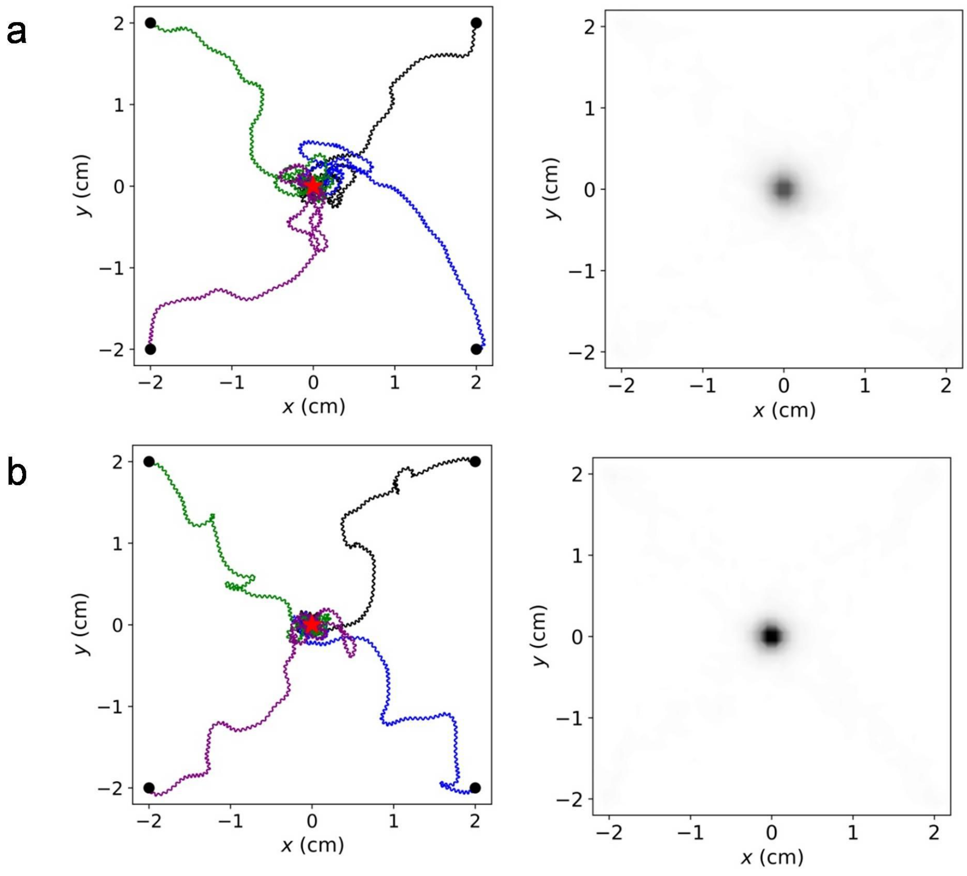

Figure S3. Comparison of the behavior generated by the random-walk model *elegans* and complete chemotaxis behavior including the biased random walk. (**a**) Behavior generated by the random-walk model *elegans*. (**b**) Complete chemotaxis behavior including the biased random walk. Left panels are four randomly selected tracks. The black dots and red stars represent the initial locomotion positions and the NaCl peaks, respectively. Right panels show density reached by all tracks at each position (n=100). Time spent at each position as a fraction of total time is indicated by the darkness of that position; frequently visited positions are darker.

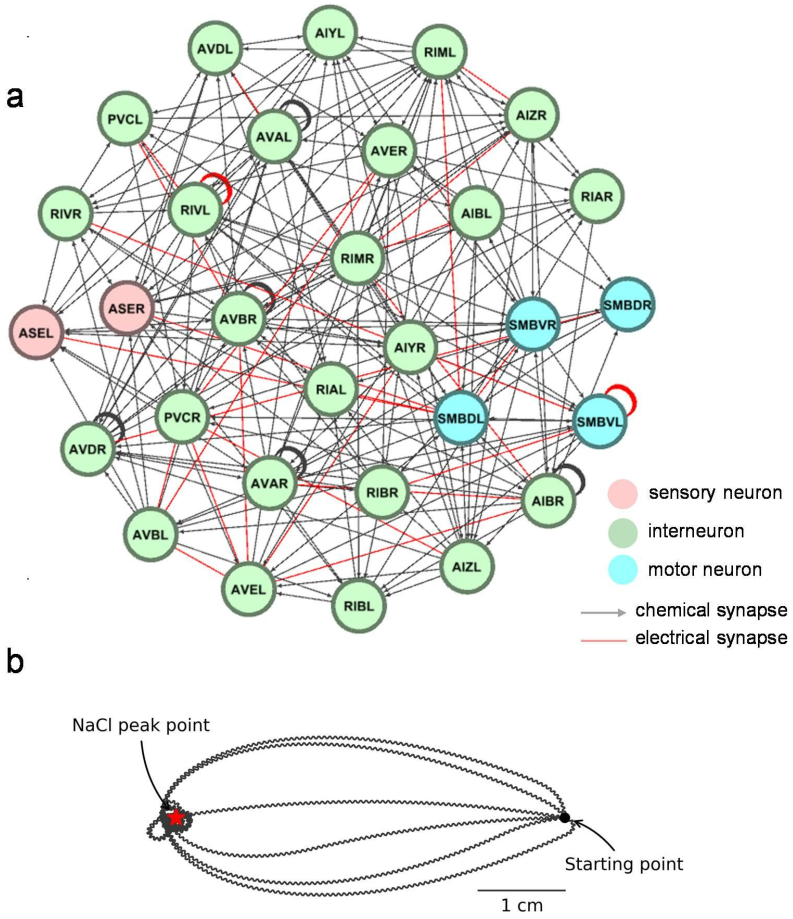

Figure S4. A random network and its locomotion tracks. (**a**) The architecture of a random network. The neurons and the total number of connections were the same as that in Fig. 8, and the connection structure was randomly generated. (**b**) Several tracks generated by this random network after its parameters were evolved by the GA for the purpose of chemotaxis.
